# Can medical assistants help improve family medicine practices in Croatia?

**DOI:** 10.3325/cmj.2025.66.439

**Published:** 2025-12

**Authors:** Drago Baković, Vanja Pintarić Japec, Anita Struški, Ino Protrka, Juraj Jug

**Affiliations:** 1 University of Zagreb School of Medicine, Zagreb, Croatia; 2 Health Center Zagreb – Center, Zagreb, Croatia; 3 Health Center Zagreb – West, Zagreb, Croatia

## Abstract

**Aim:**

To evaluate whether adding a medical assistant (MA) during team substitutions improves workload, access to care, preventive activities, and staff-reported working conditions in Zagreb family medicine practices.

**Methods:**

This case-control study involved 60 family medicine practices: 30 with an integrated MA during summer vacation team substitutions and 30 controls. Patient contacts, task distribution, and health care professionals' feedback were assessed. The MAs involved were medical or nursing students in their final years, who undertook non-medical tasks under supervision by health care staff.

**Results:**

Teams with an MA demonstrated a significant 10.55% increase in total patient contacts, responded to significantly more patient emails and phone calls, and performed significantly more preventive and chronic disease panels. Furthermore, MAs in these teams resolved up to 39% of consultations without the need for patients to directly contact health personnel. Healthcare professionals in these teams reported improved job satisfaction, reduced work-related stress, and better overall working conditions than the control group.

**Conclusion:**

Integrating an MA during team substitutions is a promising strategy for enhancing operational efficiency and improving health care standards in family medicine practices. MAs have the potential to address administrative challenges and promote patient-centered care.

In recent years, an elevated number of consultations in family practices has intensified the pressure on family physicians (FPs) (1). Administrative workload is one of the main contributors to burnout syndrome and stress at work among FPs (2-4). In many countries, administrative burden has been alleviated by expanding the team with medical or non-medical staff and by delegating tasks (4-6). Reception workers, medical assistants, and other non-medical staff are frequent team members, and the necessary competencies, education, and tasks they perform in practices differ from country to country (7,8). Non-medical professionals known as scribes can take on tasks such as entering data into documentation during a clinical encounter under supervision by a physician (4,5,9-12). In systematic reviews of burnout interventions, introducing scribes improved the team’s efficiency, reduced administrative burden, and reduced burnout (4,13). Medical assistants (MAs) serve as additional support to health personnel in family medicine practices (FMPs) (14). They perform activities related to patient visit planning, manage patients' phone and email messages or in-person communication, organize documentation, and take part in some components of direct patient care (15). This team model was shown to positively affect the quality of care, patient satisfaction, and the health system as a whole (15,16)

The average number of insured persons per FMP in Croatia is 1537, while in Zagreb this number amounts to 1686 (17). In spite of these high numbers, FMPs still consist of only one nurse and one physician, without supporting administrative staff. Such FMPs are additionally burdened when another team leaves for vacations and one FMP has to take over the patients of both teams. This may impair the availability and quality of health care for patients, and put additional stress on the health personnel.

According to the data of the Croatian Medical Chamber from 2023, there is a shortage of primary health care physicians (family doctors, gynecologists, and pediatricians), and even 80% of primary care employees were highly dissatisfied with the amount of administrative work they perform, as well as the lack of time for consultations and examinations of patients (18). However, practice-level evidence from Croatia is lacking on whether introducing MAs can reduce this administrative workload and consequently increase the time available for patient consultations and examinations, particularly during team substitutions (TS). Therefore, this research was conducted to assess the effect of adding MAs to the FMP team during a TS, and whether this measure can relieve the administrative burden on Croatian FPs.

## Methods

A case-control study was performed on a convenience sample of the FMPs within the Croatian Health System in Zagreb. Data were collected between July and August 2023 in the Health Center Zagreb – Center. The analyzed FMPs deliver care for more than 110 000 people (15% of the city's population). Each FMP team was assigned one MA. The MA staff assigned to the research group were final-year medical or nursing students, contracted and paid as student workers via the student employment service, with costs covered by the Health Center Zagreb – Center. They performed exclusively non-medical tasks, such as making appointments for the patients, preparing chronic therapy prescriptions (but could not issue them without the certification of health personnel), giving non-medical advice by phone or e-mail, filling in various administrative forms under supervision by health personnel, preparing electronic health records (EHR) for e-consultation with the physician, emailing documentation with findings to the EHR, preparing referrals according to the submitted findings, etc. Before starting to work in the practices, the MAs underwent training at the health center. They also signed a GDPR declaration, which obliged them to protect the confidentiality of patients’ data .

The study encompassed 60 FMPs, with 30 practices being allocated an MA for the duration of TS. A FP was included if it managed more than 3000 patients and if it consented to integrate an MA into their team. The remaining 30 FMPs that did not add an MA to the team served as a control group. Cases and controls were matched in terms of location, number of patients, and age groups in care.

The work performed in FM in Croatia is partly reimbursed through 179 diagnostic-therapeutic procedures (DTPs) divided into four levels (depending on the equipment and additional training of the staff needed for their implementation) determined by the income model from 2013 (Croatian Health Insurance Institute) (19).

The study and control groups were compared in terms of the number of daily contacts with patients, face-to-face (FTF) consultations (including consultations in the office, the first, control, and extended examinations, and in-person counselling in the office), the number of telephone and email consultations, and preventive examinations using the DTPs.

We conducted a short, structured survey among physicians and nurses in the FM practices. The survey consisted of 7 questions (in the Croatian language) rated on a 5-point Likert scale (from 1 to 5), capturing perceived working conditions, break use, stress level, sufficiency of consultation/examination time, ability to perform trained duties, administrative workload, and time available for preventive activities. All invited respondents completed the questionnaire (100% response). The survey questions are shown in Supplemental Table 1(Supplementary Table 1). This study was approved by the Ethics Committee of the Health Center Zagreb – Center (251-510-03-20-23-21).

### Statistical analysis

The normality of the distribution was tested with the Kolmogorov-Smirnov test. Differences between continuous variables were evaluated with a *t* test, while differences between categorical variables were assessed with a χ^2^ test. Data are expressed as mean ± standard deviation (SD), and percentages. The correlation analysis was performed with the Pearson correlation test and linear regression analysis (the share of average work done by an MA was analyzed as a dependent variable). A *P* value <0.05 was considered significant. The analysis was performed with Statistica v. 12.0 (TIBCO Software Inc., Palo Alto, CA, USA).

## Results

The FPs included in the study cared for a total of 115 022 insured persons (9624 persons younger than 18 years, 72 683 persons 19-64 years of age, and 26 510 persons aged 65 and over). During TSs, FP teams made 124 898 contacts with patients (35 503 e-mails, 40 831 phone calls, and 27 667 FTF consultations). Teams with an MA earned 25.31% more through DTPs per team (€1629 ± 694 vs €1300 ± 653, *P* = 0.008) and had 10.55% more contacts with patients (1093 ± 353 vs 989 ± 364 contacts, *P* = 0.114). Teams with an MA also answered 37.58% more e-mails (343 ± 198 vs 249 ± 165, *P* = 0.006) and performed 43.81% more telephone calls (133 ± 123 vs 92 ± 83, *P* = 0.037), but the groups did not significantly differ in the number of performed FTF consultations (239 ± 99 vs 222 ± 110, *P* = 0.361; 7.92% more in the teams with an MA). Teams with an MA performed five times more preventive panels and about twice as many chronic disease panels than teams without an MA (Table 1). MAs had 39% ± 10% of contacts with patients per time without the need for the patient to directly contact the nurse or physician. The number of panels performed was significantly higher in teams with an MA (27 vs 14 teams, χ^2^ = 13.02; *P* < 0.001).

**Table 1:** Differences between family medicine teams with and without medical assistants (MAs)*

Patient characteristics	Teams with an MA N = 30	Control group N = 30	P
Age (years)			
0-18	151.67 ± 72.94	140.26 ± 60.36	0.359
19-64	1102.61 ± 246.87	1078.19 ± 249.81	0.599
>65	394.59 ± 147.08	390.69 ± 138.72	0.884
Share of patients >65 y (%)	23.18 ± 10.99	24.70 ± 9.21	0.419
The total number of patients in both teams	3339.17 ± 305.98	3260.93 ± 397.27	0.396
**Team substitution (TS)**			
Team substitution duration (working days)	18.03 ± 2.52	18.00 ± 2.89	0.946
Diagnostic-therapeutic procedures (DTP)			
Total value of DTPs done per time during TS in one team (€)	1629.53 ± 694.92	1300.37 ± 652.62	0.008
Maximum DTPs which could be charged per time during TS (€)	1651.18 ± 320.95	1625.30 ± 338.83	0.668
Share of total DTPs done from the limited amount during TS (%)	98.90 ± 37.72	78.80 ± 29.96	<0.001
**Contacts**			
Total number of contacts	1092.98 ± 353.39	988.65 ± 363.87	0.114
The average number of contacts in one team per day	60.14 ± 16.01	54.46 ± 15.87	0.053
The average number of contacts in both teams per day	120.29 ± 17.14	108.92 ± 17.58	0.014
**E-mail contacts**			
Total number of e-mails	342.65 ± 197.61	249.07 ± 165.43	0.006
The average number of e-mails in one team per day	19.00 ± 10.22	14.07 ± 9.05	0.006
The average number of e-mails in both teams per day	38.00 ± 14.79	28.14 ± 15.38	0.014
**Telephone consultations**			
Total number of telephone consultations	214.20 ± 115.35	241.15 ± 184.61	0.339
The average number of telephone consultations in one team per day	11.73 ± 5.72	13.35 ± 9.88	0.275
The average number of telephone consultations in both teams per day	23.47 ± 10.47	26.71 ± 17.54	0.389
Total number of consultations performed over the telephone	132.82 ± 122.73	92.35 ± 83.55	0.037
The average number of consultations performed over the phone in one team per day	7.52 ± 6.65	5.01 ± 4.16	0.014
The average number of consultations performed over the phone in both teams per day	15.06 ± 12.08	10.04 ± 7.62	0.057
Face-to-face (FTF) consultations			
Total number of FTF consultations	239.35 ± 99.32	221.77 ± 110.17	0.361
The average number of FTF consultations in one team per day	13.22 ± 5.19	12.11 ± 5.41	0.251
The average number of FTF consultations in both teams per day	26.44 ± 7.67	24.21 ± 7.86	0.270
Panels			
Preventive panels during TS per team	10.18 ± 11.17	1.88 ± 3.67	<0.001
Chronic disease panels during TS per team	22.47 ± 13.23	13.27 ± 13.35	<0.001

*Data are shown as means ± standard deviations.

The amount of work performed by MAs was correlated with the age of patients in the practice (r = 0.373; *P* = 0.042), that is, there was more total work in the FMP (r = 0.412; *P* = 0.024). A larger amount of work performed by MAs was significantly correlated with the average number of FTF consultations per day (r = 0.529; *P* = 0.003) and the number of contacts with insured persons (r = 0.355; *P* = 0.05). The average number of telephone consultations (r = 0.164; *P* = 0.385) and the number of answered email messages (r = 0.063; *P* = 0.741) were not correlated with the amount of work performed by MAs. Linear regression analysis showed that the number of performed preventive and chronic panels was an independent predictor of the share of work performed by MAs. Also, a higher share of patients older than 65 years and a larger number of FTF consultations in one team per day were associated with an increase in the amount of work done by MAs (Table 2).

**Table 2:** Linear regression analysis assessing the predictors of the average share of total work done by MAs*

	Beta	Standard error beta	b	Standard error beta	P
Intercept			−0.453	2.999	0.881
Share of patients >65 y	0.303	0.098	0.227	0.073	0.003
Preventive panels in one team per time during team substitution (TS)	0.267	0.093	0.196	0.068	0.006
Chronic disease panels in one team per time during TS	0.224	0.107	0.139	0.066	0.041
The average number of contacts in one team per day	0.145	0.207	0.075	0.106	0.485
The average number of e-mails in one team per day	0.044	0.149	0.035	0.120	0.770
The average number of face-to face consultations in one team per day	0.386	0.125	0.612	0.197	0.003

*R = 0.795; R^2^ = 0.632; adjusted R^2^ = 0.590; F = 15.173; *P* < 0.001.

Both physicians and nurses who worked in teams with an MA were significantly more satisfied with the working conditions (4.47 ± 0.72 vs 2.73 ± 1.21, *P* < 0.001), used the planned rest break more often (3.86 ± 1.43 vs 2.42 ± 1.54, *P* < 0.001), and self-reported lower subjective stress (3.25 ± 1.23 vs 4.29 ± 1.01, *P* < 0.001). Teams with an MA believed they were more available for consultations and examinations of patients (3.68 ± 1.06 vs 2.04 ± 0.92, *P* < 0.001), were less burdened by administration (3.93 ± 1.05 vs 2.71 ± 1.13, *P* < 0.001), and had more time to deal with the work for which they were educated (3.22 ± 1.43 vs 4.29 ± 1.06, *P* < 0.001), which was specifically related to preventive activities (3.49 ± 1.24 vs 1.73 ± 0.83, *P* < 0.001) (Supplemental Figure 1(Supplementary Figure 1)).

## Discussion

In this study, MAs significantly alleviated the administrative burden of health personnel in FMPs during TSs, solving as much as 39% of patient consultations without the need for patients to directly contact the health personnel. While adding an MA to the FM team increased the number of total contacts with patients, and email and phone consultations, it also increased the number of provided preventive services. To our knowledge, this is the first case-control study from Croatia involving MAs, although case-control studies including MAs have been performed elsewhere (20).

The high share of consultations solved by MAs without the need for the patient to directly contact the physician or nurse reflects a high administrative burden in FMPs in Croatia. This finding is supported by earlier research (21) showing that FPs in Croatia spend 1/3 of their time on administrative work, with nurses' time being almost completely burdened by the administration. Also, a more recent analysis by Jug et al (22) showed an increase in an already high average daily number of administrative procedures after the COVID-19 pandemic in 2022 compared with 2019. In a 2020 Canadian survey, physicians of all specialties spent 10.6 hours per week on administrative tasks, with 38% deemed unnecessary (23).

Our study showed a correlation between the share of work done by MAs and an increase in the number of FTF consultations per day. Even though almost 8% more FTF consultations were found in FMPs with MAs, this difference was not statistically significant. However, linear regression analysis indicated that greater involvement by MAs can reduce the administrative burden, allowing physicians more patient time. Greater involvement of MAs was also positively correlated with more interactions between FMPs and patients, which indicates that MAs can contribute to easier access to care. Previous research found that additional team members enabled physicians to provide more services (7,16,23). Dai et al showed that physician assistants (PAs) had a stronger impact than nurse practitioners (NPs), possibly because PAs perform more substitutive than supplemental tasks (24). Not every FMP will benefit the same from an MA. Namely, while our study showed that the between-group difference in FTF consultations was not significant, a higher share of contacts handled by the MA was positively correlated with more FTF consultations per day and more total patient contacts. Therefore, the effect of adding an MA to FMPs on accessibility and scope of practice varies depending on the characteristics of the FMP, the characteristics of the physician, the physicians' readiness to delegate their tasks, and the characteristics of MAs and their previous training (7,15,16). Our findings support the thesis that MAs' work will more positively affect the FMPs caring for an older population since we observed a higher share of work done by MAs in FMPs with a higher percentage of patients older than 65 years. This finding can be explained by older patients more often needing chronic therapy prescriptions and referrals, or other administrative-related consultations.

Accessibility indicates quality in primary care (25,26). An almost 11% increase in the number of contacts with patients in teams with an MA in our study also shows that FMPs in Croatia cannot handle all patient health requests during TSs without supporting administrative staff or other measures. Due to the lack of physician workforce in primary care (18), this problem cannot be solved by increasing the number of health personnel. Team-based care is an effective strategy to respond to the increase in patient volume and to the undersupply of primary care physicians (24,27).

The addition of an MA to FMPs in this study significantly increased preventive activities. Also, the higher share of work done by MAs was correlated with an increase in performed preventive panels and chronic disease panels. Therefore, even adding an MA to an FMP can help increase preventive activities by the team, especially if the MA performs a higher share of total work. Other studies showed that expanded MA roles can improve preventive care and reduce costs by decreasing complications and hospital readmissions (16,28). In Croatia, preventive and chronic disease panels were formed to facilitate the management of chronic patients, and are recognized as quality indicators in primary care by the national health insurance agency (29).

Our study showed significantly higher satisfaction with working conditions in FMPs with an MA, indicating that MAs effectively reduced the workload on health personnel. FMPs with an MA also reported more frequent use of breaks and lower stress levels. Ramanathan et al also reported that health personnel working with upskilled administrative staff reported lower levels of stress, reduced workload, and greater work satisfaction (8). Physicians experience high levels of stress and increased workload, both globally and in Croatia. A national cross-sectional survey from 2017 showed that 63% of Croatian physicians met the burnout criteria, high emotional exhaustion or depersonalization, with an increased risk in primary and tertiary care (30). Systemic pressures, staff shortages, increased workload for remaining physicians, and increasing administrative demands further exacerbate the situation (31). In this context, empowering nurses and MAs to perform most tasks can reduce the administrative burden on physicians and improve process and quality-of-care indicators (16). Although this study did not address burnout *per se*, it shows that physicians with less administrative workload reported lower subjective stress levels and greater job satisfaction. Furthermore, an international systematic review suggests that physician burnout is associated with reduced productivity, including higher sick-day absence, reduced work capacity, and a higher intention to leave clinical practice, with considerable heterogeneity in the evidence (32). Longitudinal data from primary care further suggest that burnout predicts physicians’ leaving clinical practice over subsequent years (33).

Additionally, in our study, FMPs with an MA reported more time for consultations and physical examinations of patients and were less burdened by administration. Reducing the administrative burden and increasing the time available for patient examinations underlines the contribution of MAs to the quality of provided health care, and is in line with our previously reported objective findings.

The study's findings may have limited generalizability beyond Zagreb's health care system due to differences in infrastructure, demographics, and policies. The observational design may lead to selection bias, as MAs worked only in FMPs with physicians open to team-based care. The study focuses on short-term outcomes, not capturing long-term impacts on health care delivery, patient outcomes, and provider burnout. Additionally, results pertain to TS conditions where one physician covered two FMPs, which may not apply to standard FMP operations.

Our results may inform future health policies, showing that integrating an MA into an FMP can improve health care efficiency by enhancing working conditions, reducing stress, and allowing health care workers to focus on their duties. However, the administrative burden on health care workers can also be relieved by eliminating non-essential tasks and simplifying and supporting the remaining administration (5,21). This approach is crucial for reducing burnout among health care workers.
