# Supplementary material for: Can medical assistants help improve family medicine practices in Croatia?
Source: Croat Med J. 2025 Dec;66(6):429–35. doi: 10.3325/cmj.2025.66.439 (PMC12835998; doi:10.3325/cmj.2025.66.439)

Supplementary Figure 1

Average answers to the questions asked in a conducted survey on physicians and nurses with and without an administrator. All differences between teams were statistically significant. C = control group, A = with administrator.

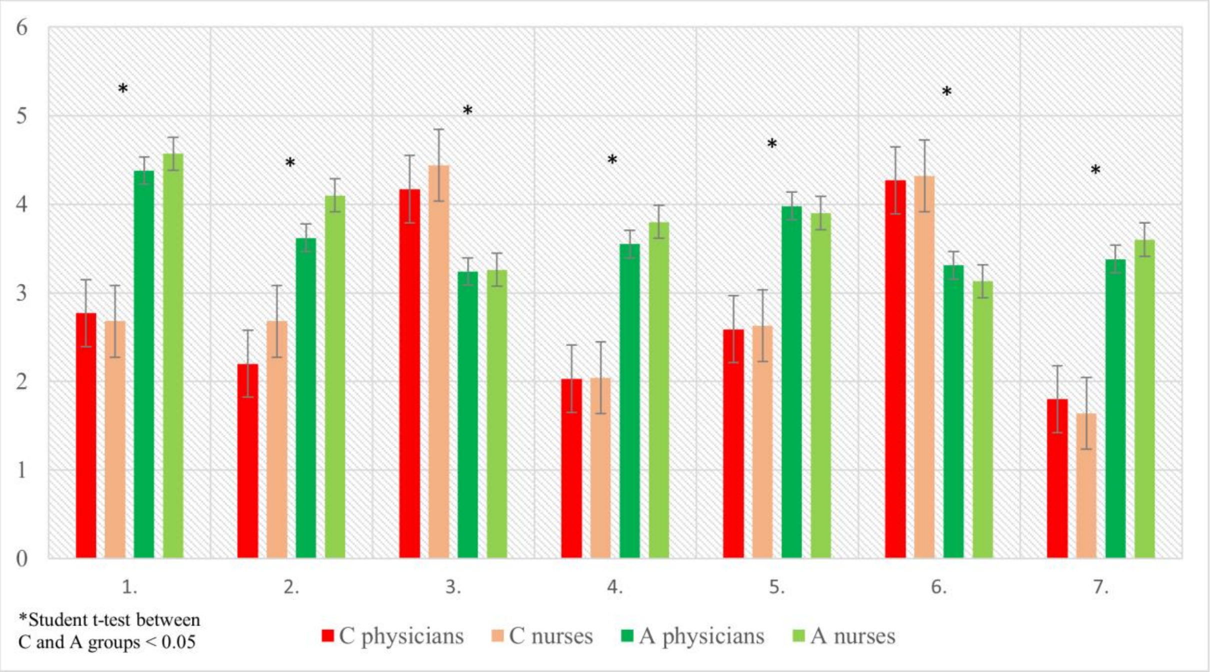

Supplement: Supplementary Figure 1 [file CroatMedJ_66_s001.pdf]
