# Supplementary material for: Can medical assistants help improve family medicine practices in Croatia?
Source: Croat Med J. 2025 Dec;66(6):429–35. doi: 10.3325/cmj.2025.66.439 (PMC12835998; doi:10.3325/cmj.2025.66.439)
Supplement: Supplementary Table 1 [file CroatMedJ_66_s002.pdf]

Supplementary Table 1

Survey questions.

Questions:
1. How satisfied are you with the working conditions during the team exchange during July / August 2023?
2. How often did you use the rest break (stipulated by the collective agreement) during the team exchange during July / August 2023?
3. How would you rate the level of stress at work during the team exchange during July / August 2023?
4. How would you assess the sufficiency of consultation time and patient examinations during the team exchange during July / August 2023?
5. How often were you able to engage in the work for which you were educated during the team exchange during July / August 2023?
6. How do you rate the administrative workload during the team exchange during July / August 2023?
7. How do you rate the time you had to engage in preventive activities during the team exchange during July / August 2023?
